# Problem drinking among Flemish students: beverage type, early drinking onset and negative personal & social consequences

**DOI:** 10.1186/s12889-018-5120-7

**Published:** 2018-02-12

**Authors:** Sara De Bruyn, Edwin Wouters, Koen Ponnet, Joris Van Damme, Lea Maes, Guido Van Hal, and the Task Force substance use in Flemish universities and colleges

**Affiliations:** 1 Department of Sociology, University of Antwerp, Sint-Jacobstraat 2, B-2000 Antwerp, Belgium; 2 Department of Communication Studies, University of Antwerp, Antwerp, Belgium; 3 Department of Communication Studies, IMEC-MICT, Ghent University, Ghent, Belgium; 4 Association for Alcohol and other Drug problems, Brussels, Belgium; 5 Department of Public Health, Ghent University, Ghent, Belgium; 6 Department of Epidemiology and Social Medicine, Social Epidemiology and Health Policy, University of Antwerp, Antwerp, Belgium

**Keywords:** Alcohol, Consequences, University and college students, Core alcohol and drug survey, Early age drinking, Beverage type

## Abstract

**Background:**

Although alcohol is socially accepted in most Western societies, studies are clear about its associated negative consequences, especially among university and college students. Studies on the relationship between alcohol-related consequences and both beverage type and drinking onset, however, are scarce, especially in a European context. The aim of this research was, therefore, twofold: (1) What is the relationship between beverage type and the negative consequences experienced by students? and (2) Are these consequences determined by early drinking onset? We will examine these questions within the context of a wide range of alcohol-related consequences.

**Methods:**

The analyses are based on data collected by the inter-university project ‘Head in the clouds?’, measuring alcohol use among students in Flanders (Belgium). In total, a large dataset consisting of information from 19,253 anonymously participating students was available. Negative consequences were measured using a shortened version of the Core Alcohol and Drug Survey (CADS_D). Data were analysed using negative binomial regression.

**Results:**

Results vary depending on the type of alcohol-related consequences: Personal negative consequences occur frequently among daily beer drinkers. However, a high rate of social negative consequences was recorded for both daily beer drinkers and daily spirits drinkers. Finally, early drinking onset was significantly associated with both personal and social negative consequences, and this association was especially strong between beer and spirits drinking onset and social negative consequences.

**Conclusions:**

Numerous negative consequences, both personal and social, are related to frequent beer and spirits drinking. Our findings indicate a close association between drinking beer and personal negative consequences as well as between drinking beer and/or spirits and social negative consequences. Similarly, early drinking onset has a major influence on the rates of both personal and social negative consequences. The earlier students started drinking, the more negative consequences they experienced during college or university. Several (policy) interventions are discussed. This study is the first to incorporate detailed information on both beverage type and drinking onset, and its associated negative consequences, as measured by the CADS_D, in a large student population.

**Electronic supplementary material:**

The online version of this article (10.1186/s12889-018-5120-7) contains supplementary material, which is available to authorized users.

## Background

Despite the fact that alcohol is a socially accepted drug in most Western societies, there is widespread empirical evidence of its associated negative consequences [1]. According to the World Health Organization, the problematic use of alcohol remains one of the five most important causes of disease, disability and death across the globe [1]. A staggering 5.9% of all deaths worldwide are caused by harmful alcohol use, rendering alcohol misuse as a recognized public health problem [1]. The harmful use of alcohol is especially a problem for young adults, such as university and college students, as the university or college experience is often characterized by high levels of substance use and more problematic alcohol use [2–7]. Moreover, students systematically overestimate the amount of alcohol needed to produce certain negative consequences, such as vomiting, which may in turn lead to heavier drinking [8]. This high prevalence of alcohol misuse among students makes it a crucial subject for research.

Alcohol-related negative consequences should not be underestimated. First of all, alcohol consumption can lead to a number of immediate health consequences affecting the drinker, such as (short term) physical discomfort (e.g., hangover, nausea and amnesia), but also (long term) health conditions such as neuropsychiatric conditions, gastrointestinal diseases, and cancers [1]. In addition to these health risks, students can suffer from school-related consequences as well. The National Institutes of Health (NIH) indicates that about 1 in 4 students in the US encounters academic problems as a consequence of their drinking (e.g. missing classes, attaining lower grades) [3].

Besides consequences for the individual, alcohol use can also harm other people in cases of aggression, assault, car accidents, or property damage. A study by Hingson et al. [9] estimated that 10.5% of fulltime students who followed a 4-year programme were injured because of alcohol use, 12% were hit or assaulted by another drinking student and 2% experienced an alcohol-related sexual assault or date rape. In 2005, 29.2% of 18-24 year old students drove under the influence [10], exponentially increasing the risk of having an accident [11]. The harm to other people and to society as a whole can be quantified in what is known as social cost. The related social cost in the whole of Belgium, where this study was performed, was estimated to be € 4.2 billion in 2013 [12]. On a European level it was estimated to be € 125 billion in 2003 [1, 13]. In conclusion, it is clear that alcohol misuse causes a major burden on society in terms of health, social and economic outcomes.

Despite the widespread empirical evidence on the negative consequences of alcohol misuse, the available studies do have some shortcomings. First of all, both beverage type and drinking onset seem to be important predictors. Indeed, some studies have indicated that beer or spirits drinking, as well as starting to drink at a younger age, are associated with alcohol-related problems or injuries [14–20]. However, these studies either focused on beverage type or drinking onset and thus did not incorporate both to produce a comprehensive picture of the variables. Furthermore, they often focus on just one or a few type(s) of negative consequences (e.g., injuries to the drinker). Key in doing research on this topic, however, is that different types of consequences need to be taken into account as alcohol (mis)use can affect both the drinkers themselves and other people. We believe more research is needed with respect to beverage type and drinking onset in relation to negative alcohol-related consequences, since policies and practices that regulate the sale or the use of alcohol are often beverage and/or age specific. Secondly, the vast majority of studies focus on a US context [16–19]. It is uncertain, however, whether results from these studies could be extrapolated to a European or a Belgian context given the differences with regard to the school system, alcohol policies and related student drinking behaviour. US student life is characterized by living in dormitories and a legal drinking age of 21, whereas the Belgian situation is characterized by lower rates of students living in dormitories and a legal drinking age of 16 for fermented and 18 for distilled alcoholic beverages [21, 22]. We will address the shortcomings of current literature by using a large Flemish dataset, including both beverage type and drinking onset as well as a range of several negative consequences. A growing body of research has focused on developing and applying scales to measure alcohol-related negative consequences among young people [8, 23–33]. We used a shortened version of the consequence scale of the Core Alcohol and Drug Survey (CADS_D) [33, 34].

The primary aim of this research is to investigate two key questions: 1) What is the relationship between beverage type and the negative consequences experienced by students? and 2) Are these consequences determined by early drinking onset? Based on the available studies, we would expect beer and spirits drinking to be associated with alcohol-related consequences. Moreover, we hypothesize that the younger students start drinking, the more consequences they will experience as a student. This research thus expands on current literature by thoroughly investigating the association between beverage types (beer, wine, other non-distilled beverages, and spirits) and early drinking onset; and a range of negative consequences experienced by students, as measured by the CADS_D. To the best of our knowledge, this study is the first to examine the negative consequences of alcohol use with this amount of detail in a large student population. By obtaining more detailed results, we hope these can be of particular importance to both micro-level health care workers and macro-level policy makers in order to prevent alcohol-related negative consequences among students.

## Methods

### Procedure

This retrospective study is based on a substance use data collection among university and college students in Flanders (Belgium) in 2013, entitled ‘Head in the clouds?’. The survey was made available for four to six weeks in eleven participating universities and colleges between February and April 2013. Students were invited by e-mail and other methods (e.g., student magazine) to participate anonymously. No reminder was sent [2]. The study was performed according to the ethical standards of the American Psychological Association and was approved by the Ethics Committee of the Ghent University Hospital (EC UZG 2013/065).

Five colleges were excluded from the sample because of their low response rate (< 3.5%). The remaining six institutions were located in different regions of Flanders (Ghent, Leuven, Hasselt and Antwerp). In total 19,253 college students (22.1% response rate) were included in the sample. This response rate is similar to other large-scale online surveys among college students [35, 36]. The sample consisted of 35.67% men (*n* = 6867) and 64.33% women (*n* = 12.386). Mean age of participants was 21.12 years (SD = 3.25). We compared sex and institution distributions between our sample and the total student population enrolled in the six participating institutions [37]. As shown in Table 1, Chi-squared tests indicated that our sample had a higher proportion of women (*χ*^*2*^ = 673.07, *p* <  0.001) and students from the university of Ghent, ‘KHLimburg’ College and ‘Group T’ College (*χ*^*2*^ = 1456.99, *p* <  0.001).

**Table 1:** Chi-square difference tests between our sample and the population based on the stratification criteria institution and sex

Stratification criteria	Total population (*N* = 87,119) % (n)	Sample (*n* = 19,253) % (n)	*P*-value (*χ*^*2*^ test)
Institution			< 0.001
University of Antwerp	13.05% (11,366)	9.85% (1896)	
University of Ghent	30.56% (26,627)	37.30% (7181)	
University of Leuven	35.34% (30,785)	26.95% (5189)	
‘KdG’ College	11.89% (10,359)	11.68% (2249)	
‘KHLimburg’ College	6.34% (5521)	10.84% (2087)	
‘Group T’ College	2.82% (2461)	3.38% (651)	
Sex			< 0.001
Men	44.97% (39,176)	35.67% (6867)	
Women	55.03% (47,943)	64.33% (12,386)	

### Measures

*Demographic information*: sex (man; woman), age (in years), living situation (at home; on campus; living independently); attending university/college.

*Frequency of alcohol use.* This variable was measured for each beverage type separately. Respondents were asked how often they drink beer, wine, other non-distilled beverages such as Martini and sherry, and spirits during the academic year, the exam period and holidays. Response categories were 1 = *never*, 2 = *less than or once a month*, 3 = *less than once a week but more than once a month*, 4 = *once a week*, 5 = *more than once a week but less than daily*, 6 = *daily*. As a categorical variable, multiple dummy codes were created to include in the model. Since the intention of this research is to give a representative image of alcohol use among students during the academic year, we use the variable ‘frequency of alcohol use during the academic year’. Both exam and holiday periods are outside of our scope of interest since drinking behaviour can differ in these periods.

*Drinking onset.* Respondents needed to indicate at what age they drank alcohol for the first time (either a sip or a whole drink). This open-ended question was asked for each beverage type. During analysis, responses were coded in six categories: *less than 15 years old, 15, 16, 17, 18* and *more than 18 years old.* Similar to the previous variable, multiple dummy codes were created.

*Negative consequences of alcohol use* were measured using the CADS [38]. Participants were asked how often they had experienced a list of 19 consequences (e.g., got into an argument or fight) as a consequence of their drinking or drug use during the last year. The answer categories were ‘none’, ‘one’, ‘two’, ‘three to five’, ‘six to nine’ and ‘10 or more times’. The ranges were recoded using mid-points of the categories, respectively 0, 1, 2, 4, 7.5, and 11.25 times for the upper category (10 times plus half range to midpoint of adjacent category) [39]. The complete list of consequences is presented in Additional file 1. In a previous paper [40], we investigated the psychometric properties of the CADS focusing on its factor structure, as well as its validity and reliability. This resulted in a shortened version of the CADS (CADS_D) which indicated a two-factor structure, identifying personal negative consequences and social negative consequences. Although the convergent validity of the factor ‘social negative consequences’ could be improved, CADS_D was concluded to be a valid and reliable instrument to screen for alcohol-related consequences among college students. Factor loadings of the items of the two scales were all close to or larger than 0.50, no cross-loadings between indicators were present and the covariance of the two factors was lower than 0.80. The factor personal consequences had a Cronbach’s Alpha of 0.78 and the factor social consequences had one of 0.66. The factor ‘personal negative consequences’ referred to consequences experienced by the drinkers themselves and contained three items: had a hangover, became nauseated or vomited, missed a class. The factor ‘social negative consequences’ referred to consequences that not only affected the drinker, but also other people. This factor contained four items: got into an argument or fight, been criticized by someone I know, done something I later regretted, been hurt or injured.

### Analytic strategy

The two factors (personal and social negative consequences), each measured as the sum of the underlying items, were used as dependent variables in the regression models. The complete list of consequences is thus only used for descriptive purposes. The independent variables of interest consisted of drinking frequency and drinking onset. Both variables were measured for different beverage types. We controlled for age, sex, institution and living situation. Frequency of other substance use (stimulant medication, tranquillizers/sedatives, cannabis, ecstasy, amphetamines and cocaine) were also used as control variables to ensure that consequences of alcohol use were not related to other drugs.

We used negative binomial regression to test whether drinking frequency and drinking onset for several beverage types were associated with the personal and social drinking consequences. Prior research has shown that the use of negative binomial regression is the best method for analysing these overdispersed count data [41, 42]. Since we were only interested in the consequences students experienced as a result of their past year’s alcohol use, separate regressions were conducted for each type of beverage after excluding the lowest frequency from the sample. For example, when investigating the frequency of beer drinking in relation to both personal and social consequences, we excluded the lowest frequency of beer drinking (which represented participants who had never drunk beer before or had not drunk beer in the past 12 months). Frequencies of other beverage types were used as additional control variables. The same process was repeated for each beverage type.

Negative binomial regression analyses were conducted with IBM SPSS Statistics 22.

## Results

### Descriptive statistics

In total, 96.05% of the participants stated that they had used alcohol before, of which 97.04% had used alcohol in the past 12 months. Half of these students (51.66%; *n* = 9111) indicated drinking beer regularly (i.e., drinking beer once a week or more). With regard to wine, non-distilled beverages and spirits, regular drinking was less prevalent than beer (22.97%, 5.32% and 14.77%, respectively). One third (35.98%; *n* = 6205) indicated drinking one beverage type regularly, 17.89% (*n* = 3085) two beverage types, 5.71% (*n* = 985) three beverage types and 1.35% (*n* = 233) all four beverage types. The majority of participants who ever drank alcohol and started drinking beer before the legal age limit of 16 years, also started drinking wine (60.99%; *n* = 6080) before the legal age of 16 years, and/or non-distilled beverages (77.04%; *n* = 6565) before 18 years and/or spirits (82.86%; *n* = 8061) before 18 years.

More drinking characteristics of the students are provided in Table 2.

**Table 2:** Drinking characteristics of the sample

	Beer (% (n))		Wine (% (n))		Non-distilled beverages (% (n))		Spirits (% (n))	
	Man	Woman	Man	Woman	Man	Woman	Man	Woman
Frequency of drinking during the academic year								
Never	19.77 (760)	80.23 (3084)	38.18 (1264)	61.82 (2047)	36.14 (3265)	63.86 (5769)	29.49 (1347)	70.51 (3221)
Less than once a month	18.85 (529)	81.15 (2278)	41.32 (2660)	58.68 (3778)	35.80 (2171)	64.20 (3894)	31.50 (2095)	68.50 (4556)
Less than once a week, more than once a month	24.75 (780)	75.25 (2372)	34.79 (1740)	65.21 (3262)	33.22 (883)	66.78 (1775)	39.47 (1932)	60.53 (2963)
Once a week	36.74 (1184)	63.26 (2039)	26.72 (637)	73.28 (1747)	35.49 (247)	64.51 (449)	46.65 (814)	53.35 (931)
More than once a week, less than daily	56.42 (2980)	43.58 (2302)	21.82 (336)	78.18 (1204)	28.77 (63)	71.23 (156)	52.72 (417)	47.28 (374)
Daily	82.01 (497)	17.99 (109)	35.42 (34)	64.58 (62)	66.67 (8)	33.33 (4)	67.57 (25)	32.43 (12)
Drinking onset before the age of 15	44.10 (2291)	55.90 (2904)	37.47 (1593)	62.53 (2658)	44.07 (383)	55.93 (486)	37.71 (408)	62.29 (674)

The top three most frequently encountered consequences of the CADS were: “*had a hangover”* (65.65%), “*became nauseated or vomited”* (56.53%) and “*missed a class”* (43.95%). Driving while intoxicated or under the influence was also reported by 8.21% of the participants. Moreover, 23.42% of the participants had not experienced any of the stated consequences during the past year. However, 19.51% of the students had encountered six or more different consequences. Participants experienced a mean number of 3.15 different types of consequences in the past year (SD = 2.82).

### Negative binomial regression analyses

The two variables ‘Personal negative consequences’ and ‘Social negative consequences’ served as dependent variables in our regression models, shown in Tables 3 and 4, respectively.

**Table 3:** Negative binomial regression of personal negative consequences with drinking frequency, sex, age, drinking onset as independent variables^a^

	Beer			Wine			Non-distilled beverages			Spirits		
	*b* (SE)	Incident rate ratio Exp (*b*)	95% CI	*b* (SE)	Incident rate ratio Exp (*b*)	95% CI	*b* (SE)	Incident rate ratio Exp (*b*)	95% CI	*b* (SE)	Incident rate ratio Exp (*b*)	95% CI
Intercept	0.78 (0.11)	2.19***	1.76-2.73	0.68 (0.11)	1.98***	1.58-2.47	0.68 (0.17)	1.98***	1.43-2.75	0.92 (0.13)	2.52***	1.97-3.21
Drinking frequency^b^												
Daily	1.30 (0.06)	3.67***	3.27-4.11	0.52 (0.13)	1.68***	1.30-2.17	−0.09 (0.55)	0.92	0.31-2.69	0.81 (0.24)	2.26***	1.40-3.63
More than once a week, less than daily	1.16 (0.03)	3.20***	3.01-3.41	0.36 (0.04)	1.43***	1.33-1.54	0.02 (0.08)	1.02	0.86-1.20	0.48 (0.05)	1.62***	1.48-1.78
Once a week	0.81 (0.03)	2.24***	2.10-2.39	0.27 (0.30)	1.31***	1.24-1.39	0.04 (0.05)	1.04	0.95-1.15	0.39 (0.03)	1.47***	1.38-1.57
Less than once a week, more than once a month	0.40 (0.03)	1.49***	1.40-1.59	0.19 (0.02)	1.21***	1.16-1.27	−0.01 (0.03)	1.00	0.94-1.05	0.27 (0.02)	1.31***	1.26-1.37
Sex^b^												
Woman	−0.07 (0.02)	0.93***	0.89-0.97	−0.09 (0.02)	0.92***	0.88-0.96	−0.08 (0.03)	0.93**	0.88-0.98	−0.13 (0.02)	0.88***	0.84-0.92
Age	−0.02 (0.01)	0.98**	0.98-0.99	−0.02 (0.00)	0.98***	0.97-0.99	−0.03 (0.01)	0.97***	0.96-0.98	−0.02 (0.00)	0.98***	0.97-0.99
Drinking onset^b^												
> 18	−0.45 (0.08)	0.64***	0.54-0.75	−0.23 (0.06)	0.79***	0.71-0.89	−0.14 (0.06)	0.87*	0.78-0.98	−0.40 (0.05)	0.67***	0.61-0.74
18	−0.38 (0.06)	0.68***	0.61-0.76	−0.10 (0.04)	0.90*	0.83-0.98	−0.05 (0.05)	0.96	0.86-1.06	−0.27 (0.04)	0.76***	0.70-0.83
17	−0.41 (0.04)	0.67***	0.62-0.72	−0.09 (0.03)	0.92*	0.86-0.98	−0.04 (0.05)	0.96	0.87-1.07	−0.16 (0.04)	0.85***	0.78-0.92
16	− 0.25 (0.03)	0.78***	0.74-0.82	− 0.02 (0.03)	0.98	0.94-1.04	0.02 (0.05)	1.02	0.92-1.13	−0.08 (0.04)	0.93	0.85-1.00
15	−0.04 (0.02)	0.96	0.91-1.01	0.06 (0.03)	1.07*	1.01-1.13	0.09 (0.06)	1.10	0.98-1.24	−0.00 (0.05)	1.00	0.91-1.09

^a^Other control variables: using tranquilizers and sedatives, stimulant medication, cannabis, amphetamines, ecstasy and cocaine; institution; living situation

^b^The reference categories for drinking frequency, sex and drinking onset are ‘less than once a month’, ‘Man’ and ‘< 15 years’ respectively

Significance levels: * *p* < 0.05; ** *p* < 0.01; *** *p* < 0.001

**Table 4:** Negative binomial regression of social negative consequences with drinking frequency, sex, age, drinking onset as independent variables^a^

	Beer			Wine			Non-distilled beverages			Spirits		
	*b* (SE)	Incident rate ratio Exp (*b*)	95% CI	*b* (SE)	Incident rate ratio Exp (*b*)	95% CI	*b* (SE)	Incident rate ratio Exp (*b*)	95% CI	*b* (SE)	Incident rate ratio Exp (*b*)	95% CI
Intercept	0.71 (0.14)	2.03***	1.53-2.69	0.60 (0.15)	1.82***	1.36-2.44	0.56 (0.22)	1.74**	1.15-2.66	1.11 (0.16)	3.04***	2.22-4.17
Drinking frequency^b^												
Daily	1.35 (0.07)	3.87***	3.39-4.42	0.50 (0.15)	1.65***	1.24-2.18	0.47 (0.59)	1.60	0.51-5.04	1.35 (0.28)	3.84***	2.24-6.60
More than once a week, less than daily	1.10 (0.04)	3.00***	2.76-3.26	0.10 (0.04)	1.11*	1.02-1.21	0.02 (0.10)	1.02	0.85-1.24	0.71 (0.05)	2.03***	1.83-2.25
Once a week	0.69 (0.04)	1.99***	1.83-2.17	0.04 (0.04)	1.04	0.97-1.12	0.10 (0.06)	1.11	0.99-1.24	0.47 (0.04)	1.60***	1.48-1.72
Less than once a week, more than once a month	0.41 (0.04)	1.50***	1.38-1.63	0.05 (0.03)	1.05	0.99-1.11	−0.08 (0.03)	0.92*	0.86-0.99	0.34 (0.03)	1.40***	1.33-1.48
Sex^b^												
Woman	−0.18 (0.03)	0.83***	0.79-0.88	−0.18 (0.03)	0.83***	0.79-0.88	−0.18 (0.03)	0.84***	0.79-0.90	−0.24 (0.03)	0.79***	0.75-0.83
Age	−0.06 (0.01)	0.94***	0.93-0.95	−0.07 (0.01)	0.94***	0.93-0.95	−0.07 (0.01)	0.93***	0.92-0.94	−0.07 (0.01)	0.93***	0.92-0.94
Drinking onset^b^												
> 18	−0.58 (0.11)	0.56***	0.45-0.70	−0.23 (0.08)	0.79**	0.68-0.92	−0.27 (0.07)	0.76***	0.66-0.87	−0.50 (0.06)	0.61***	0.54-0.68
18	−0.65 (0.08)	0.52***	0.45-0.60	−0.31 (0.05)	0.74***	0.66-0.82	−0.22 (0.06)	0.80***	0.71-0.91	−0.47 (0.05)	0.62***	0.56-0.69
17	−0.50 (0.05)	0.61***	0.55-0.68	−0.27 (0.04)	0.77***	0.71-0.83	−0.21 (0.06)	0.81***	0.72-0.92	−0.41 (0.05)	0.66***	0.60-0.73
16	−0.40 (0.03)	0.67***	0.63-0.72	−0.16 (0.03)	0.85***	0.80-0.90	−0.08 (0.06)	0.93	0.82-1.04	−0.28 (0.05)	0.75***	0.69-0.83
15	−0.13 (0.03)	0.88***	0.83-0.93	−0.08 (0.03)	0.92*	0.86-0.98	0.07 (0.07)	1.07	0.93-1.22	−0.10 (0.05)	0.90	0.81-1.00

^a^Other control variables: using tranquilizers and sedatives, stimulant medication, cannabis, amphetamines, ecstasy and cocaine; institution; living situation

^b^The reference categories for drinking frequency, sex and drinking onset are ‘less than once a month’, ‘Man’ and ‘< 15 years’ respectively

Significance levels: * *p* < 0.05; ** *p* < 0.01; *** *p* < 0.001

#### Beverage type – Drinking frequency

Negative binomial regression indicates a high incident rate ratio (IRR) of personal negative consequences for beer drinkers. As shown in Table 3, the rate is 3.67 times higher for daily beer drinkers compared with students who drink beer less than once a month. Regarding the rate of social negative consequences, data show that both daily spirits drinkers (IRR = 3.84; *p* <  0.001) as well as daily beer drinkers (IRR = 3.87; *p* <  0.001) experience strongly increased rates, as indicated in Table 4. This means that the rate of social negative consequences is 3.8 times higher among daily spirits drinkers than among students who drink spirits less than once a month, and 3.9 times higher among daily beer drinkers than among students who drink beer less than once a month. Results also indicate a significant association between wine drinking frequency and personal negative consequences, but the IRR were less strong than the IRR for beer and spirits. Associations between wine drinking frequency and social negative consequences; and non-distilled beverage drinking frequency and both personal and social negative consequences were almost all not significant, except for daily wine drinking and drinking wine more than once a week but less than daily, indicating higher rates for social negative consequences (IRR = 1.65; *p* < 0.001 and IRR = 1.11; *p* < 0.05, respectively), and drinking non-distilled beverages less than once a week but more than once a month, indicating a lower rate of social negative consequences (IRR = 0.92; *p* < 0.05).

#### Drinking onset

The age at which students started drinking alcohol is also related to negative consequences experienced. The association was especially strong between the onset of beer and spirits drinking and social negative consequences. Compared with < 15 year drinking onset, the rates for beer drinking were as follows: 0.88 (15 year olds), 0.67 (16 year olds), 0.61 (17 year olds), 0.52 (18 year olds), 0.56 (> 18 year olds). These results were all significant. Regarding spirits intake, the data were as follows: 0.90 (15 year olds), 0.75 (16 year olds), 0.66 (17 year olds), 0.62 (18 year olds), 0.61(> 18 year olds). This means that a student who started drinking spirits at the age of 19 will encounter approximately 40% lower rates of social negative consequences compared with those who started drinking spirits before the age of 15. All results were significant, except for the age category of 15 year olds. For almost all age categories, wine and non-distilled beverage drinking onset were significantly associated with rates of social negative consequences, but the associations were less strong compared to those for beer and spirits.

## Discussion

The aim of the current research was to examine the association between beverage type and early drinking onset, and the negative consequences experienced by students. Research on this topic is particularly relevant among young people and more specifically university and college students, since the university or college experience is often characterized by high levels of problematic alcohol use [2, 3].

Our analyses were based on data from the ‘Head in the clouds?’ survey, in which nearly 20,000 students from several universities and colleges in Flanders (Belgium) participated in 2013 [2]. Negative consequences were measured using the Core Alcohol and Drug Survey (CADS). Negative binomial regression was used to analyse the overdispersed count data.

The descriptive statistics make clear that 8.2% of the respondents in this sample drink/take drugs and drive (CADS does not differentiate between alcohol and other drugs). Although this percentage is lower than in the US [43], it still represents a significant problem. In Belgium, the blood alcohol concentration (BAC) limit of maximum 0.05% has been in force since 1994, and no distinction in BAC is made between young drivers and the general population. In 2015, the former Minister of Mobility proposed making a distinction in BAC between the two groups as young people are more of a risk in traffic due to their inexperience. However, no changes to the law have yet been introduced. Previous studies have made clear that lowering the BAC limit could be very effective in saving a lot of lives [9, 44, 45]. This lower limit could be applied to young people only, as is the case in some other European countries [1], or to the entire population.

Secondly, our study findings indicate that beer and spirits drinking frequency is closely related to rates of both personal and social negative consequences, with a strong association between daily beer drinking and personal negative consequences as well as between daily beer and/or spirits drinking and social negative consequences. Although more research is needed to disentangle the reasons why specific beverage types are associated with alcohol-related negative consequences, several possible explanations can be given. Firstly, previous research has indicated that beer and spirits drinkers exhibit riskier drinking patterns compared to people who drink other alcoholic beverages [20, 46–49]. Therefore, students who drink beer or spirits might be more likely to be exposed to alcohol-related risks [14]. Secondly, drinking beer and spirits could be a reflection of a specific drinking culture or a drinking lifestyle. Literature has shown that adolescent beer and spirits drinkers are often people who like to have fun, and who love to feel the effects of alcohol and to get drunk [46]. In this respect, drinking beer could be the cheapest way and spirits the fastest way to get drunk. Although this study was performed among adolescents, it is unlikely that this would differ for college and university students. Wine, on the other hand, has shown to be related to a more moderate lifestyle and is more frequently drunk at home and/or during meals [15, 20, 46]. A number of studies have shown that alcohol-related injuries were more likely to occur in public settings compared to private places [14, 15, 46]. Finally, the privileged position of beer as part of the Belgian cultural identity (acknowledged by UNESCO), and for which the legal drinking age is lower than that for spirits and non-distilled beverages, could create the perception among students that beer is safer and more socially desirable to drink compared to other alcoholic beverages. This might cause students to underestimate the risks of drinking beer. From a public health perspective, there is no reason to favour beer compared to spirits as our results indicate that they are both related to negative alcohol-related consequences.

Thirdly, our results show that the earlier students start drinking, the more negative consequences they will experience during college or university. This is especially true for young beer drinkers. This association could partly be explained by the fact that students who start drinking at an early age are more likely to be involved in frequent heavy drinking later in life, which increases the risk of negative alcohol-related consequences [18]. Another valuable explanation for this association would be that early drinkers exhibit more risk-taking behaviour in general [17, 19] and thus would be more prone to bringing themselves in risky situations when drinking alcohol. However, more research is needed to fully understand the reasons behind the association of early drinking onset and negative alcohol consequences.

Preventing alcohol misuse and negative consequences during college or university requires a comprehensive approach in which multiple parties (parents, teachers, peers, health-care workers, policy makers,…) need to be involved and which needs to start early in life, especially during adolescence. On a micro- and meso-level these research results are of particular importance to health care workers who work with adolescents and/or students as the results draw attention to the numerous risks related to excessive beer and spirits drinking, as well as to early drinking onset. Questioning patients about their drinking onset age and educating them on the alcohol-related negative consequences would be an important step in this respect [17]. Moreover, brief intervention studies could be effective in reducing negative alcohol-related consequences among college students [50, 51]. On a macro-level, policy interventions should focus on delaying the drinking onset of young people, such as increasing the legal drinking age limit. Most European countries have set the legal age limit for purchasing alcohol at 18 years [1, 52]. Some countries, such as Belgium, Denmark, Finland and Germany, however, differentiate age limits according to beverage type. Belgium, for example, has an age limit of 16 for fermented alcoholic beverages and 18 for distilled alcoholic beverages. Discussion is needed to decide whether the minimum age of 16 for fermented alcoholic beverages is still tenable. Setting a minimum age of 18 would lower the number of negative consequences experienced, would be consistent with most other European countries [1] and would create a powerful message in showing young people the risks of drinking alcohol at a young age.

Despite the widespread empirical evidence of alcohol-related negative consequences, alcohol regulation policies often have a relatively low priority on the public policy agenda [1]. Changes are desperately needed on a European level [1]. It is important to bear cultural contexts in mind and to make sure that proposed policy interventions are country specific [1]. We strongly believe that the aforementioned suggestions are feasible for implementation in Belgium and would be effective in reducing the consequences of alcohol (mis)use.

There are some limitations present in this study. Firstly, longitudinal data are not available, so the results cannot be compared over time and thus causal relationship cannot be determined. However, the negative consequences of alcohol use were measured with the question ‘How often have you experienced the following consequences as a result of your drinking during the last year’. The question thus already involves the causality we were investigating. Moreover, the association between drinking onset and alcohol-related consequences could not be interpreted in the other direction, because of the inherent chronological order of the two variables. Secondly, a significant number of negative consequences were excluded from the analyses since these were either encountered by less than 5% of participants or had loading difficulties in the factor analysis. Nonetheless, these items remain important for future studies. Thirdly, although not mentioned in the article, we also did some additional analyses in which we included the quantity of alcohol consumed on drinking days and problematic drinking behaviour such as frequency of binge drinking as control variables. However, because of the high correlation between these additional control variables and some of the other predicting factors (e.g., drinking frequency) as well as the high robustness of the initial model, we have not included these additional control variables in the model. For future studies it might be interesting to search for other factors that are associated with the dependent variable. Finally, the response rate for our sample was rather low. Although it is similar to other large-scale online surveys [35, 36], care needs to be taken in generalizing the prevalence rates to the wider student population since some groups, such as women, were over-represented in our database.

## Conclusion

The results of this study draw attention to the numerous risks related to excessive beer and spirits drinking as well as early drinking onset. To the best of our knowledge, this study is the first to incorporate detailed information on both beverage type and drinking onset and its associated negative consequences in a large student population in a European (Flemish) context. These results are especially important for both micro- (e.g. social work) and macro-level (e.g. legislative) interventions which should focus on reducing alcohol intake and delaying drinking onset age among young people as these factors are significantly related to both personal and social negative alcohol-related consequences.

## Additional file

Additional file 1: Table S1. Core Alcohol and Drug Survey – Consequences scale. This table represents the complete CADS scale, consisting of 19 negative consequences of alcohol use. (DOCX 12 kb)

## Abbreviations

BAC: Blood Alcohol Content
CADS: Core Alcohol and Drug Survey – in this research the abbreviation refers specifically to the question battery of negative consequences
CADS_D: Shortened version of the Dutch CADS
IRR: Incident rate ratio
SE: Standard error
