# Supplementary material for: Problem drinking among Flemish students: beverage type, early drinking onset and negative personal & social consequences
Source: BMC Public Health. 2018 Feb 12;18:234. doi: 10.1186/s12889-018-5120-7 (PMC5809947; doi:10.1186/s12889-018-5120-7)
Supplement: Supplementary file 1 — Core Alcohol and Drug Survey – Consequences scale. This table represents the complete CADS scale, consisting of 19 negative consequences of alcohol use. (DOCX 12 kb) [file 12889_2018_5120_MOESM1_ESM.docx]

**Table S1. Core Alcohol and Drug Survey – Consequences scale**

| **Item** |  |
| --- | --- |
| **1** | Had a hangover |
| **2** | Performed poorly on a test or important project |
| **3** | Been in trouble with police, residence hall, or other college authorities |
| **4** | Damaged property, pulled fire alarm, etc. |
| **5** | Got into an argument or fight |
| **6** | Got nauseated or vomited |
| **7** | Driven a car while under the influence (DUI) |
| **8** | Missed a class |
| **9** | Been criticized by someone I know |
| **10** | Thought I might have a drinking or other drug problem |
| **11** | Had a memory loss |
| **12** | Done something I later regretted |
| **13** | Been arrested for DWI/DUI |
| **14** | Have been taken advantage of sexually |
| **15** | Have taken advantage of another sexually |
| **16** | Tried unsuccessfully to stop using |
| **17** | Seriously thought about suicide |
| **18** | Seriously tried to commit suicide |
| **19** | Been hurt or injured |
